# Supplementary material for: Temporal partitioning and spatiotemporal avoidance among large carnivores in a human-impacted African landscape
Source: PLoS One. 2021 Sep 10;16(9):e0256876. doi: 10.1371/journal.pone.0256876 (PMC8432863; doi:10.1371/journal.pone.0256876)
Supplement: S1 File — (PDF) [file pone.0256876.s001.pdf]

S1 Survey grid summary information

Table S1.1: Summary information for the four survey grids in Ruaha-Rungwa.

	**Ruaha NP**<br>*Acacia-Commiphora*	**Ruaha NP**<br>miombo woodland	**MBOMIPA WMA**<br>*Acacia-Commiphora*	**Rungwa GR**<br>miombo woodland
**Study site details**				
PA name	Ruaha	Ruaha	MBOMIPA	Rungwa
PA designation	National Park	National Park	WMA	Game Reserve
Management authority	TANAPA[1]	TANAPA[1]	Member villages[2]	TAWA[3]
Trophy hunting permitted	No	No	Yes	Yes
Trophy hunting carried out at the time of study	No	No	No	Yes
**Camera trap survey details**				
Survey period	19 Jun - 10 Sep 2018	5 Aug - 3 Nov 2018	19 Sep - 28 Nov 2018	3 Jul - 12 Oct 2019
Survey duration (nights)	83	90	70	90
Stations	45	26	40	40
Trap nights	3,601	2,187	2,689	3,395
Average spacing	1.96 km	1.88 km	2.08 km	3.46 km
Survey area[4]	223 km^2	152 km^2	270 km^2	555 km^2

[1] Tanzania National Parks Authority

[2] The WMA is managed by an elected board called the “Authorised Association”, comprised of representatives from the 21 member villages located in the Idodi and Pawaga administrative wards of Iringa Rural District

[3] Tanzania Wildlife Management Authority

[4] Area of the minimum convex polygon around all stations (does not include buffer)
