# Supplementary material for: Temporal partitioning and spatiotemporal avoidance among large carnivores in a human-impacted African landscape
Source: PLoS One. 2021 Sep 10;16(9):e0256876. doi: 10.1371/journal.pone.0256876 (PMC8432863; doi:10.1371/journal.pone.0256876)
Supplement: S2 File — (PDF) [file pone.0256876.s002.pdf]

S2 Location and activity of community camera traps

Table S2.1: Location and activity of the 56 community camera traps, deployed in village land to the east of Ruaha NP and MBOMIPA WMA.

Village	Camera	Longitude	Latitude	Large carnivores detected	Camera make	2018						2019					
						Jun	Jul	Aug	Sep	Oct	Nov	Jun	Jul	Aug	Sep	Oct	Nov
Idodi	CCT-17-02	35.15034	-7.64235	Y	Reconyx	1	1	1	1	1	1	1	1	1	1	1	0
Idodi	CCT-17-08	35.15601	-7.62953	Y	Reconyx	1	1	1	1	1	1	1	1	1	1	1	0
Idodi	CCT-18-01	35.15811	-7.67280	Y	HCO Scout Guard	0	0	0	1	1	1	1	1	1	1	1	0
Idodi	RCP-106--	35.15607	-7.68267	N	HCO Scout Guard	1	1	1	0	0	0	0	0	0	0	0	0
Isele	CCT-17-05	35.35852	-7.37561	Y	Reconyx	1	1	1	1	1	1	0	0	0	0	0	0
Isele	CCT-17-09	35.34554	-7.37561	Y	Reconyx	1	1	1	1	1	0	0	0	0	0	0	0
Isele	CCT-17-24	35.34396	-7.37921	N	HCO Scout Guard	1	0	0	0	0	0	0	0	0	0	0	0
Isele	CCT-17-37	35.34396	-7.37921	Y	HCO Scout Guard	0	1	1	1	1	1	0	0	0	0	0	0
Isele	CCT-18-21	35.35356	-7.36744	N	Cuddeback	0	0	0	0	0	0	0	0	0	0	1	0
Isele	CCT-18-22	35.35040	-7.35769	N	Cuddeback	0	0	0	0	0	0	0	0	0	0	1	0
Kinyika	CCT-17-16	35.40663	-7.32134	Y	Reconyx	1	1	0	1	0	0	0	0	0	0	0	0
Kinyika	CCT-17-38	35.40953	-7.31277	Y	HCO Scout Guard	0	1	1	1	1	1	1	1	0	0	0	0
Kinyika	CCT-18-10	35.40957	-7.31277	N	Cuddeback	0	0	0	0	0	1	0	0	0	0	0	0
Kinyika	CCT-18-23	35.41363	-7.31180	N	Cuddeback	0	0	0	0	0	0	0	0	0	0	1	0
Kitisi	CCT-17-27	35.15161	-7.69181	Y	HCO Scout Guard	1	1	1	1	1	1	1	1	0	0	0	0
Kitisi	CCT-17-34	35.14868	-7.70122	Y	HCO Scout Guard	1	1	0	0	0	0	0	0	0	0	0	0
Kitisi	CCT-18-11	35.14508	-7.69897	Y	Cuddeback	0	0	0	0	0	0	1	1	1	1	1	0
Kitisi	CCT-18-18	35.15145	-7.68975	Y	Cuddeback	0	0	0	0	0	0	1	1	1	1	1	0
Kitisi	CCT-18-26	35.15145	-7.68975	Y	Cuddeback	0	0	0	0	0	0	0	0	0	0	1	0
Kisilwa	CCT-18-01	34.96141	-7.93679	Y	Cuddeback	0	0	0	1	1	1	1	1	1	1	1	1
Kisilwa	CCT-18-02	34.95568	-7.94571	Y	Cuddeback	0	0	0	1	1	1	1	1	1	1	1	1
Kisilwa	CCT-18-04	34.96589	-7.92630	Y	HCO Scout Guard	0	0	0	1	1	1	1	1	1	1	1	1
Kisanga	CCT-17-10	35.46862	-7.26873	Y	Reconyx	1	1	1	1	1	1	0	0	0	0	0	0
Kisanga	CCT-17-25	35.46744	-7.26157	Y	HCO Scout Guard	1	1	1	1	1	1	1	1	1	1	1	1
Kisanga	CCT-18-11	35.46591	-7.25899	Y	HCO Scout Guard	0	0	0	1	0	0	0	0	0	0	0	0
Kisanga	CCT-18-12	35.46274	-7.25460	N	Cuddeback	0	0	0	0	0	0	1	1	1	0	0	0
Luganga	CCT-18-08	35.42846	-7.48337	Y	Cuddeback	0	0	0	1	1	1	1	1	1	1	1	1

Luganga	CCT-18-14	35.41808	-7.48063	N	HCO Scout Guard	0	0	0	1	1	1	1	1	1	1	1	1
Mafuluto	CCT-17-12	35.34613	-7.50591	Y	Reconyx	1	1	1	1	1	1	1	1	1	1	1	1
Mafuluto	CCT-17-29	35.35599	-7.50629	Y	HCO Scout Guard	1	1	1	1	1	1	0	0	0	0	0	0
Mafuluto	CCT-17-36	35.36992	-7.51287	Y	HCO Scout Guard	0	0	0	1	1	1	0	0	0	0	0	0
Magombwe	CCT-17-17	35.35799	-7.39066	Y	Reconyx	1	1	1	1	1	1	0	1	1	1	1	1
Magombwe	CCT-17-28	35.34626	-7.38422	Y	HCO Scout Guard	1	1	1	1	1	1	1	1	1	1	1	0
Magombwe	CCT-18-15	35.35676	-7.38935	N	HCO Scout Guard	0	0	0	1	1	1	0	0	0	0	0	0
Mahuninga	CCT-17-18	34.96635	-7.91420	Y	HCO Scout Guard	1	1	1	0	0	0	0	0	0	0	0	0
Mahuninga	CCT-17-19	34.97984	-7.88327	Y	HCO Scout Guard	1	1	1	0	0	0	0	0	0	0	0	0
Mahuninga	CCT-18-03	34.97188	-7.90242	Y	HCO Scout Guard	0	0	0	1	1	1	1	1	1	1	1	1
Makifu	CCT-17-11	35.02943	-7.85269	Y	Reconyx	1	1	1	1	1	1	1	1	1	1	1	0
Makifu	CCT-17-32	35.02088	-7.82928	Y	HCO Scout Guard	1	1	1	1	1	1	1	1	1	1	1	0
Makifu	CCT-18-02	35.02336	-7.83232	Y	HCO Scout Guard	0	0	0	1	1	1	1	1	1	1	1	0
Malinzanga	CCT-17-01	35.31456	-7.56639	Y	Reconyx	1	1	1	1	1	1	1	1	1	1	1	1
Malinzanga	CCT-17-36	35.30836	-7.56968	N	HCO Scout Guard	1	1	1	0	0	0	0	0	0	0	0	0
Malinzanga	CCT-18-08	35.31647	-7.56163	Y	HCO Scout Guard	0	0	0	1	1	1	1	1	1	1	1	0
Malinzanga	RCP-NEW-3	35.31595	-7.55106	Y	HCO Scout Guard	1	1	1	1	1	1	1	1	1	1	1	1
Mapogoro	CCT-18-07	35.09116	-7.79963	Y	HCO Scout Guard	0	0	0	1	1	1	1	1	1	1	1	1
Mapogoro	CCT-18-09	35.08677	-7.80631	Y	Cuddeback	0	0	0	0	0	1	0	0	0	0	0	0
Mboliboli	CCT-18-05	35.62420	-7.36832	N	Cuddeback	0	0	0	1	1	1	1	1	1	1	1	1
Mboliboli	CCT-18-06	35.62579	-7.37670	N	Cuddeback	0	0	0	0	1	0	0	0	0	0	0	0
Mboliboli	CCT-18-13	35.63313	-7.37713	N	HCO Scout Guard	0	0	0	0	1	1	1	0	0	0	0	0
Mboliboli	CCT-18-25	35.60953	-7.36586	N	Cuddeback	0	0	0	0	0	0	0	0	0	0	1	1
Mbugani	CCT-18-03	35.62559	-7.36450	N	Cuddeback	0	0	0	1	1	1	1	1	1	0	0	1
Mbugani	CCT-18-04	35.63512	-7.36379	N	Cuddeback	0	0	0	1	1	1	1	1	1	0	1	1
Mbugani	CCT-18-12	35.63026	-7.35585	N	HCO Scout Guard	0	0	0	1	1	1	1	1	1	0	1	1
Tungamalenga	CCT-17-14	35.01892	-7.79996	Y	Reconyx	1	1	1	1	1	1	1	1	1	1	1	1
Tungamalenga	CCT-17-35	35.03171	-7.82388	Y	HCO Scout Guard	1	1	1	1	1	1	0	0	0	0	0	0
Tungamalenga	CCT-18-16	35.01892	-7.79996	N	Cuddeback	0	0	0	0	0	0	1	1	1	0	0	0
