# Supplementary material for: Temporal partitioning and spatiotemporal avoidance among large carnivores in a human-impacted African landscape
Source: PLoS One. 2021 Sep 10;16(9):e0256876. doi: 10.1371/journal.pone.0256876 (PMC8432863; doi:10.1371/journal.pone.0256876)

S5 Maps of large carnivore capture events

Figure S5.1: The number of unique large carnivore capture events at each camera station. Captures were considered unique if they occurred more than 30 minutes after the previous capture of the same species at the same station, unless the animals captured could be confidently distinguished as different individuals. Circles are scaled according to the number of captures at that station; note that a unique scale is used for each species, dependent on the maximum number of captures at a single station of that species.

(A) Leopard

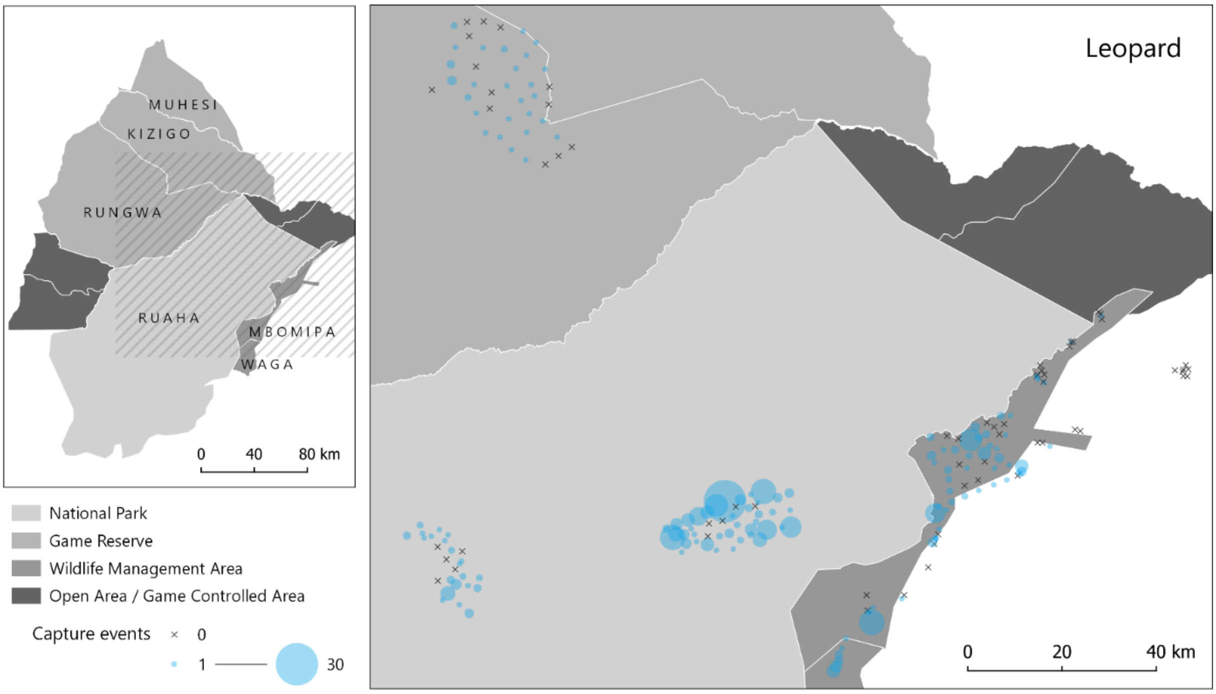

(B) Lion

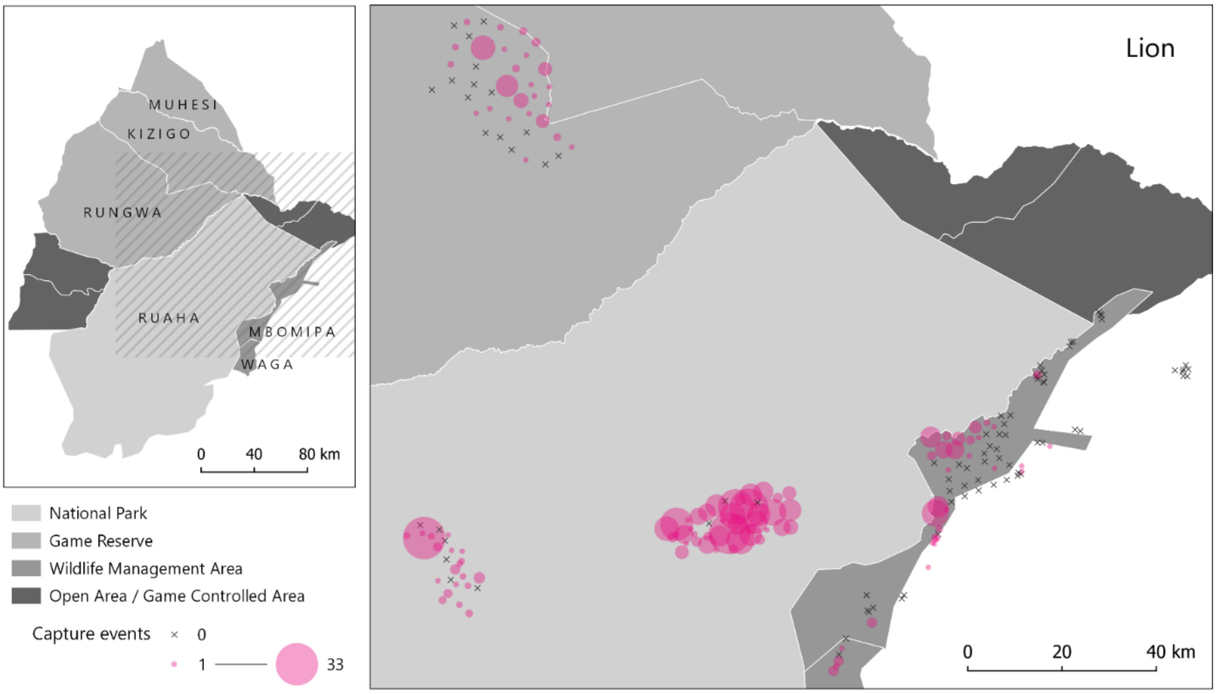

(C) Spotted hyaena

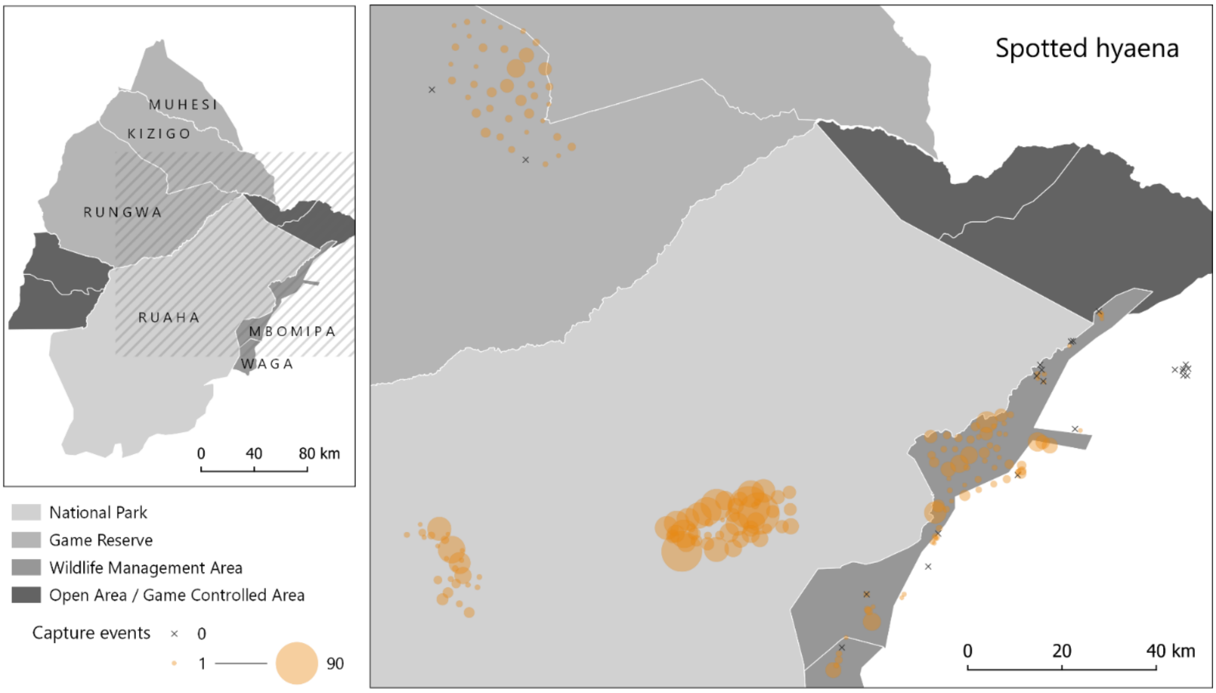

(D) Striped hyaena

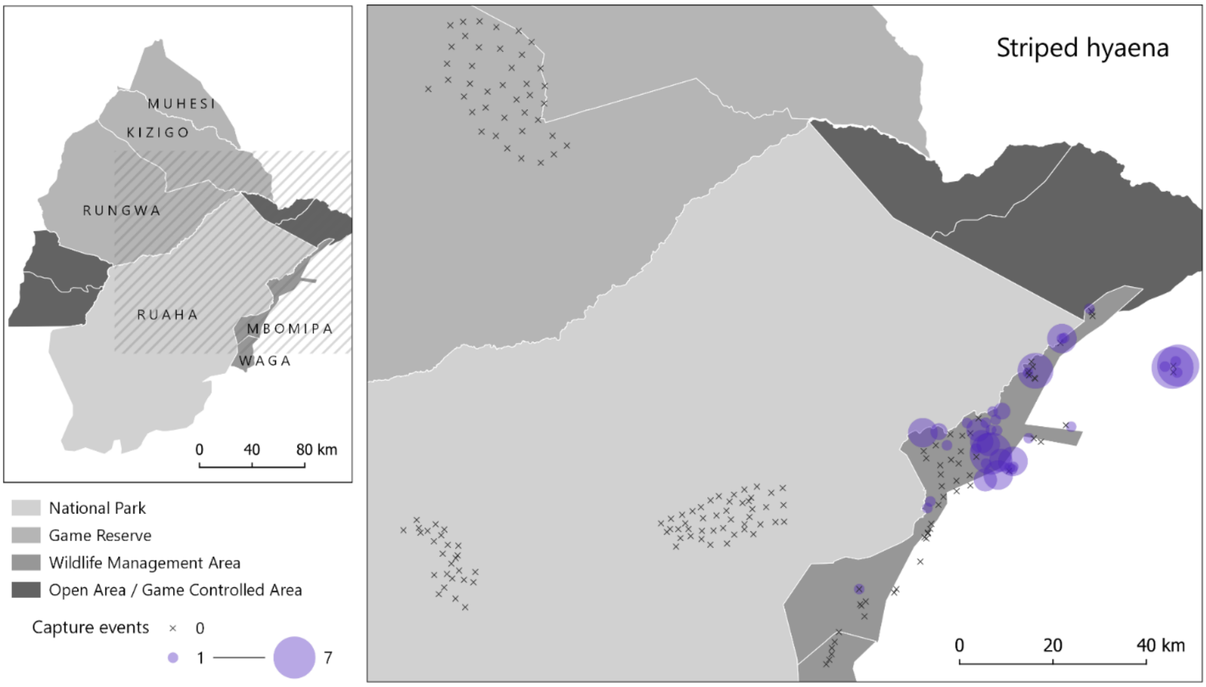

(E) African wild dog

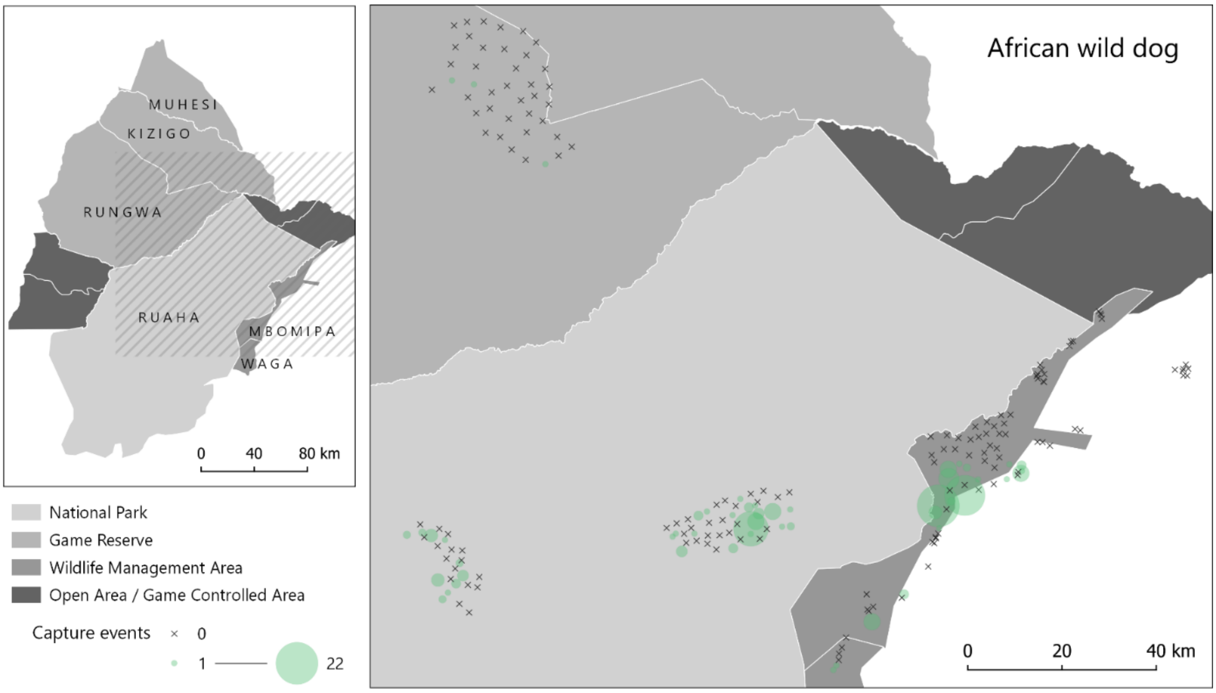

(F) Cheetah

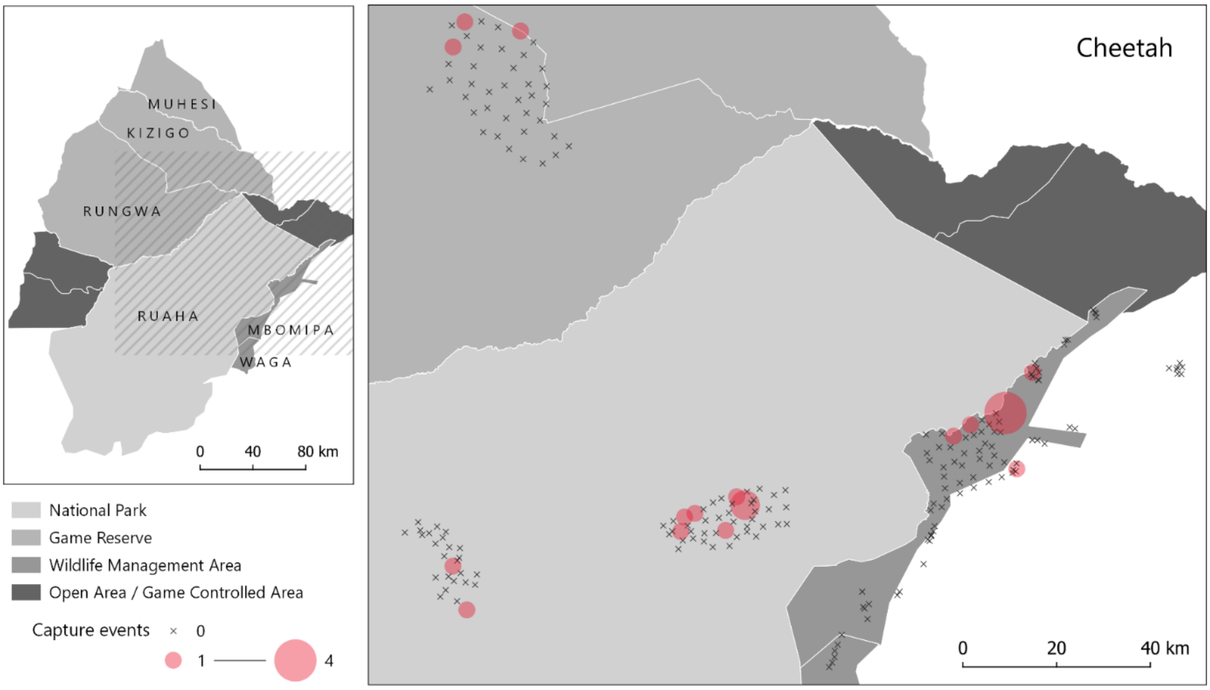

Supplement: S5 File — (PDF) [file pone.0256876.s005.pdf]
