# Supplementary material for: Temporal partitioning and spatiotemporal avoidance among large carnivores in a human-impacted African landscape
Source: PLoS One. 2021 Sep 10;16(9):e0256876. doi: 10.1371/journal.pone.0256876 (PMC8432863; doi:10.1371/journal.pone.0256876)

S6 Plots of within- and between-species activity overlap

Within-species overlap

Figure S6.1: Activity pattern overlap for each large carnivore species between the different survey sites, produced by fitting a kernel density function to the capture data for each species. Areas shaded in grey correspond to the coefficient of overlap (Δ) for the species between the two sites of interest. Each study site is represented by a three letter code: RNP = Ruaha NP *Acacia-Commiphora*, MIO = Ruaha NP miombo woodland, WMA = MBOMIPA WMA *Acacia-Commiphora*, RGI = Rungwa GR miombo woodland, CCT = village land (community camera-trapping programme). Activity patterns marked with a single asterisk are significantly different (*; p < .05), and those marked with two asterisks are highly significantly different (**; p < .001).

(A) Leopard

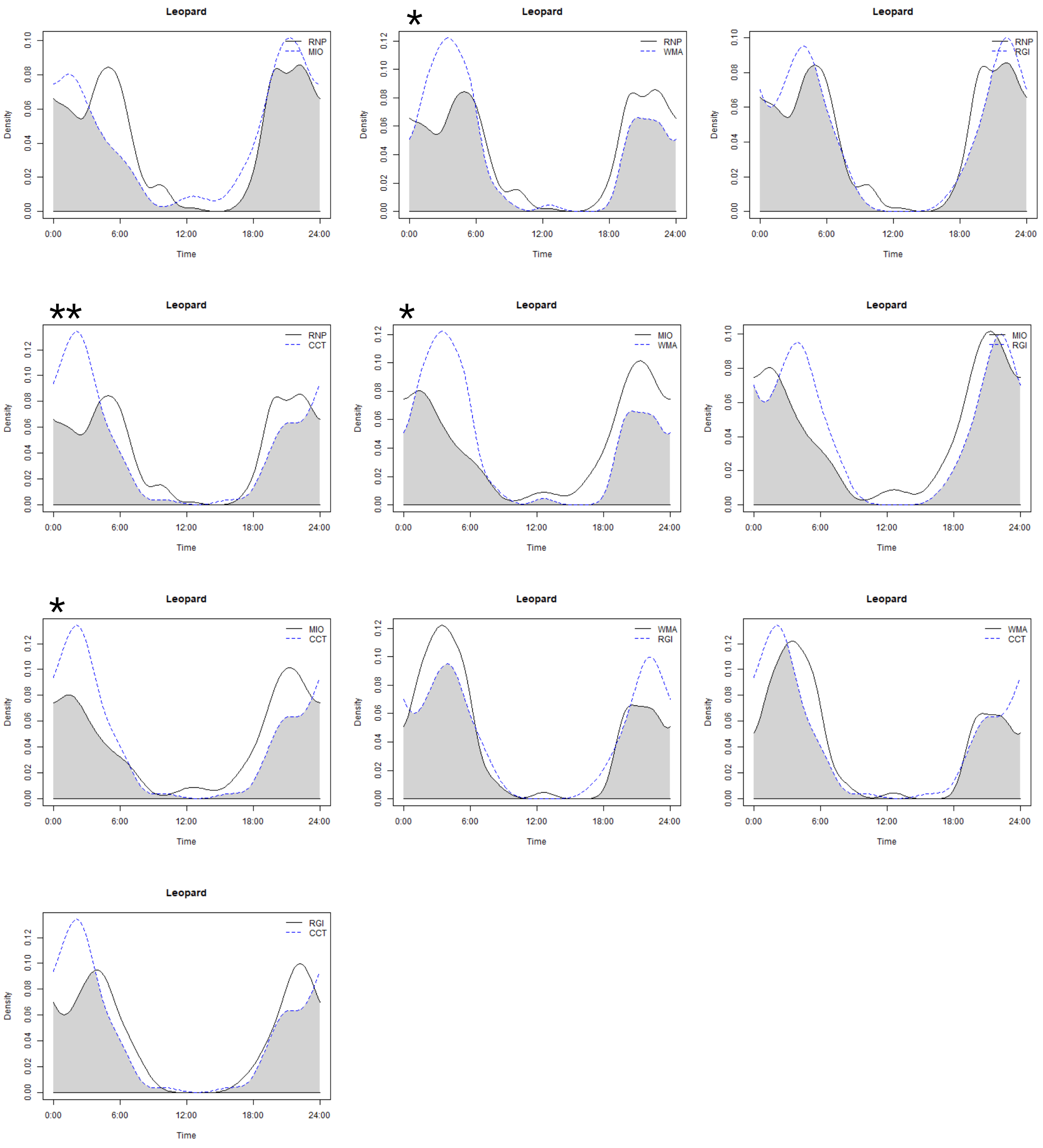

(B) Lion

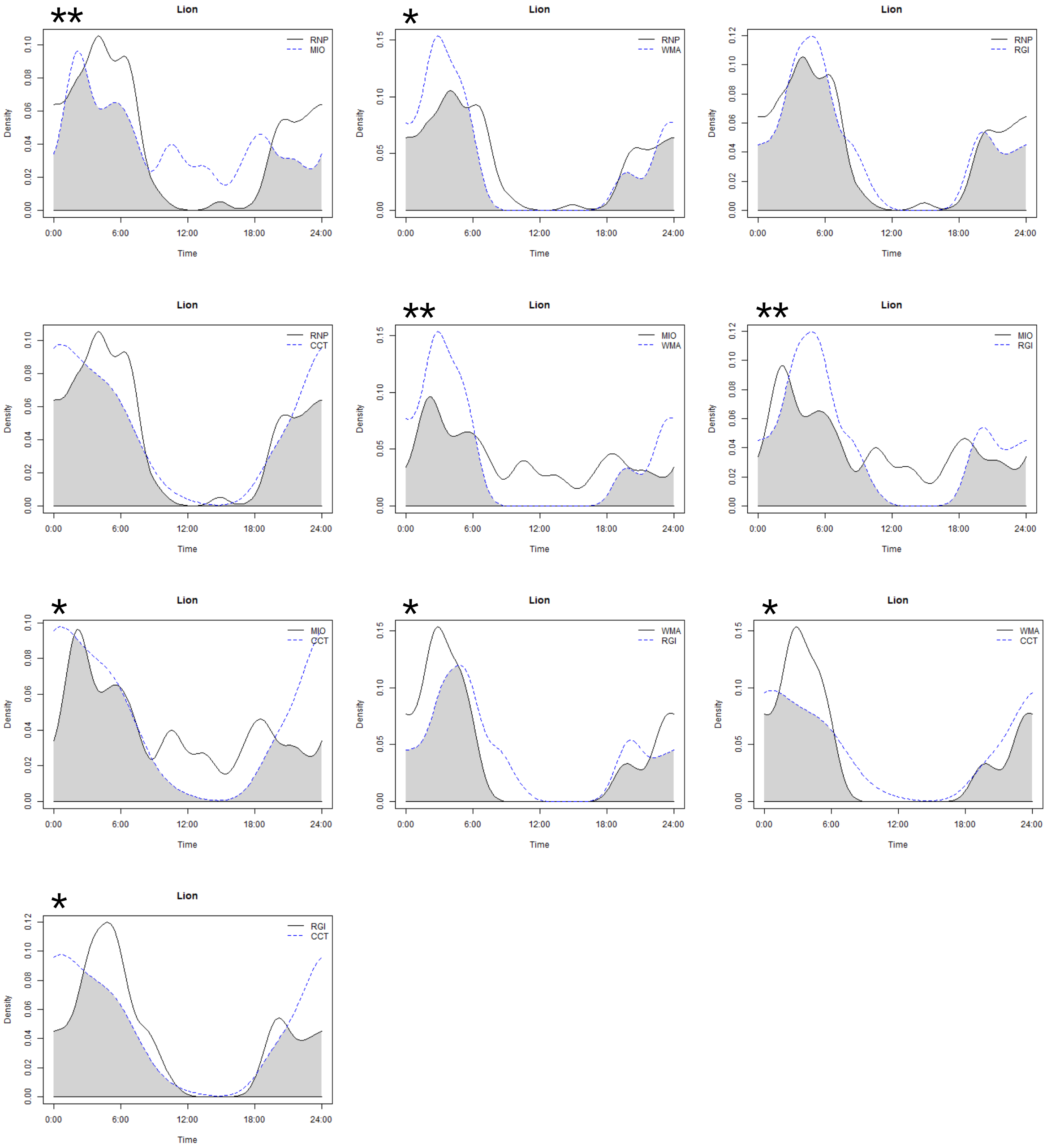

**
Lion
RNP
MIO
Density
0.00
0.02
0.04
0.06
0.08
0.10
0:00
6:00
12:00
18:00
24:00
Time
*
Lion
RNP
WMA
Density
0.00
0.05
0.10
0.15
0:00
6:00
12:00
18:00
24:00
Time
Lion
RNP
RGI
Density
0.00
0.02
0.04
0.06
0.08
0.10
0.12
0:00
6:00
12:00
18:00
24:00
Time
Lion
RNP
CCT
Density
0.00
0.02
0.04
0.06
0.08
0.10
0:00
6:00
12:00
18:00
24:00
Time
**
Lion
MIO
WMA
Density
0.00
0.05
0.10
0.15
0:00
6:00
12:00
18:00
24:00
Time
**
Lion
MIO
RGI
Density
0.00
0.02
0.04
0.06
0.08
0.10
0.12
0:00
6:00
12:00
18:00
24:00
Time
*
Lion
MIO
CCT
Density
0.00
0.02
0.04
0.06
0.08
0.10
0:00
6:00
12:00
18:00
24:00
Time
*
Lion
WMA
RGI
Density
0.00
0.05
0.10
0.15
0:00
6:00
12:00
18:00
24:00
Time
*
Lion
WMA
CCT
Density
0.00
0.05
0.10
0.15
0:00
6:00
12:00
18:00
24:00
Time
*
Lion
RGI
CCT
Density
0.00
0.02
0.04
0.06
0.08
0.10
0.12
0:00
6:00
12:00
18:00
24:00
Time

(C) Spotted hyaena

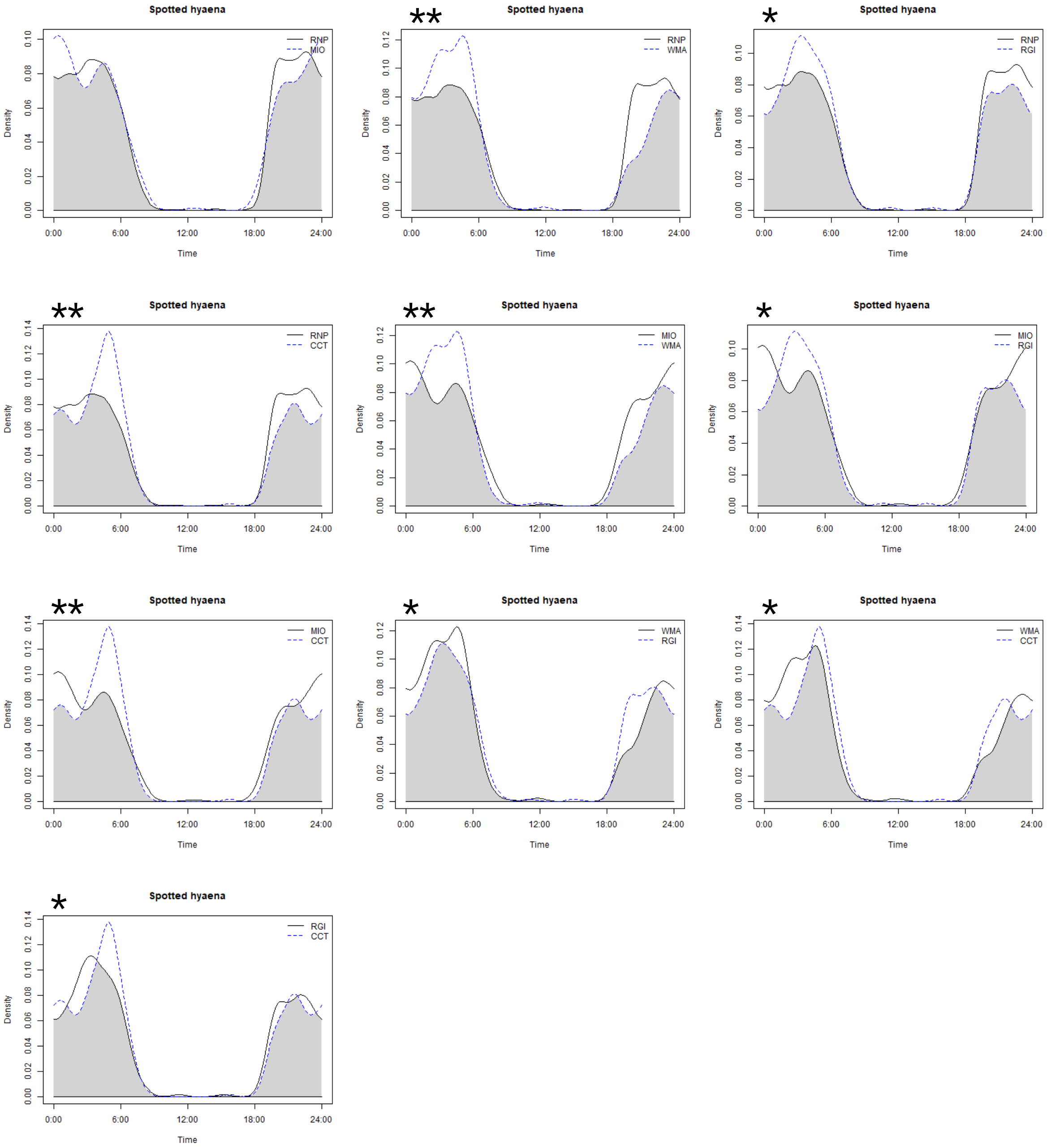

(D) Striped hyaena

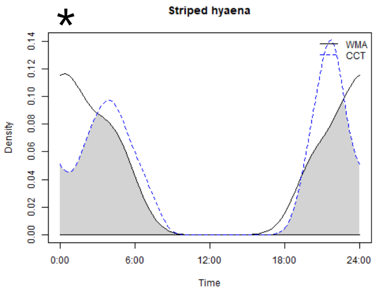

(E) African wild dog

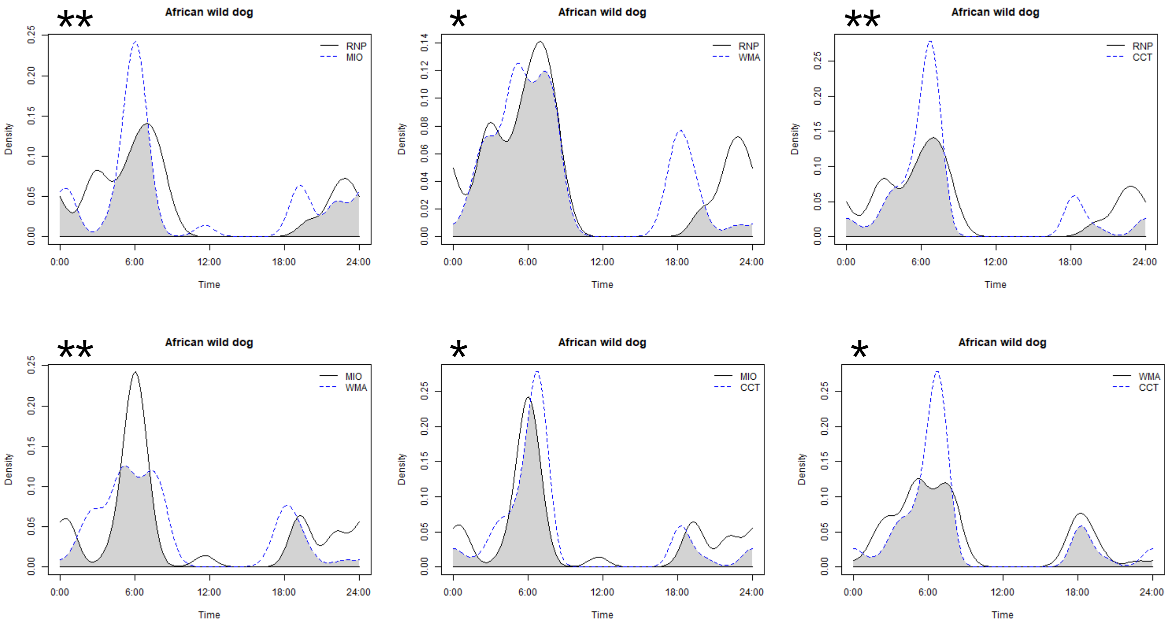

Between species overlap

Figure S6.2: Activity pattern overlap for each large carnivore species or sex pair (for lion and leopard only) across the five survey sites and using data from all sites combined, produced by fitting a kernel density function to the capture data for each species. Areas shaded in grey correspond to the coefficient of overlap (Δ) for that species pair. Each study site is represented by a three letter code: RNP = Ruaha NP *Acacia-Commiphora*, MIO = Ruaha NP miombo woodland, WMA = MBOMIPA WMA *Acacia-Commiphora*, RGI = Rungwa GR miombo woodland, CCT = village land (community camera-trapping programme). Activity patterns marked with a single asterisk are significantly different (*; $p < .05$), and those marked with two asterisks are highly significantly different (**; $p < .001$).

(A) Leopard – Lion

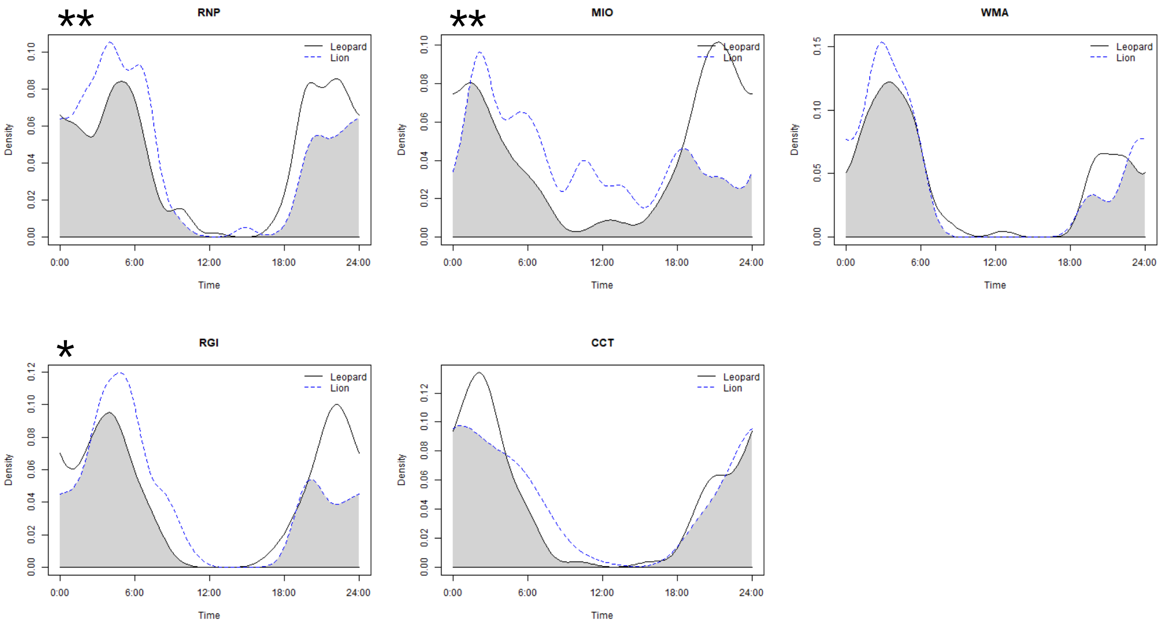

(B) Leopard – Spotted hyaena

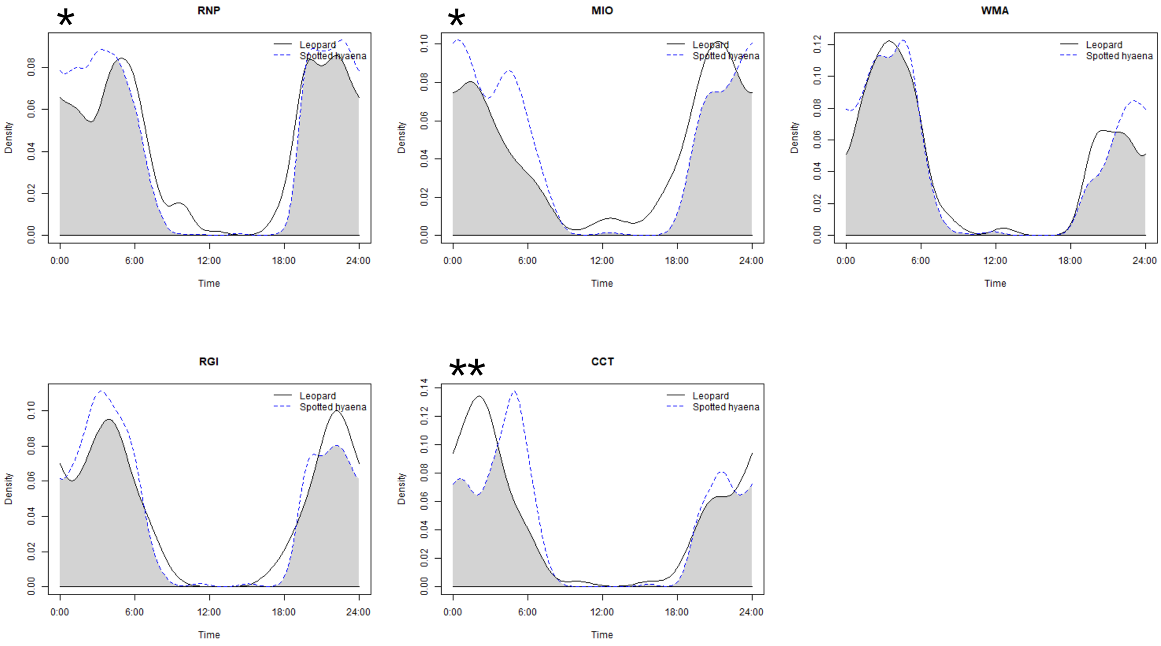

(C) Leopard – Striped hyaena

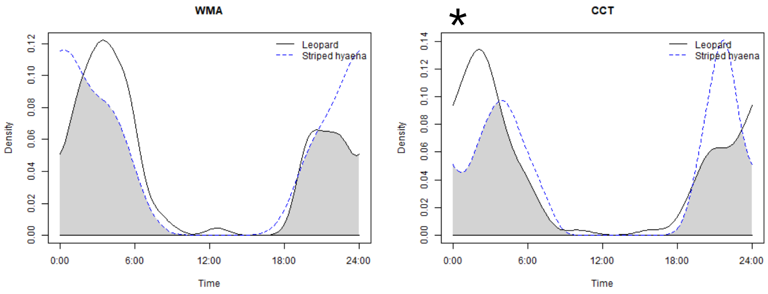

(D) Leopard – African wild dog

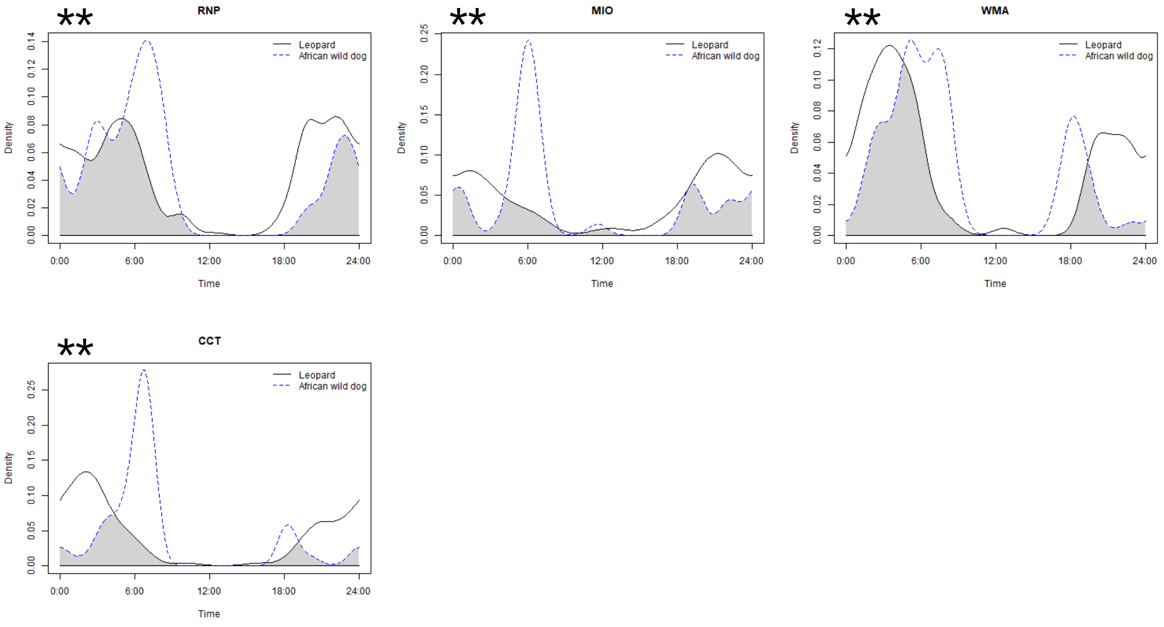

(E) Lion – Spotted hyaena

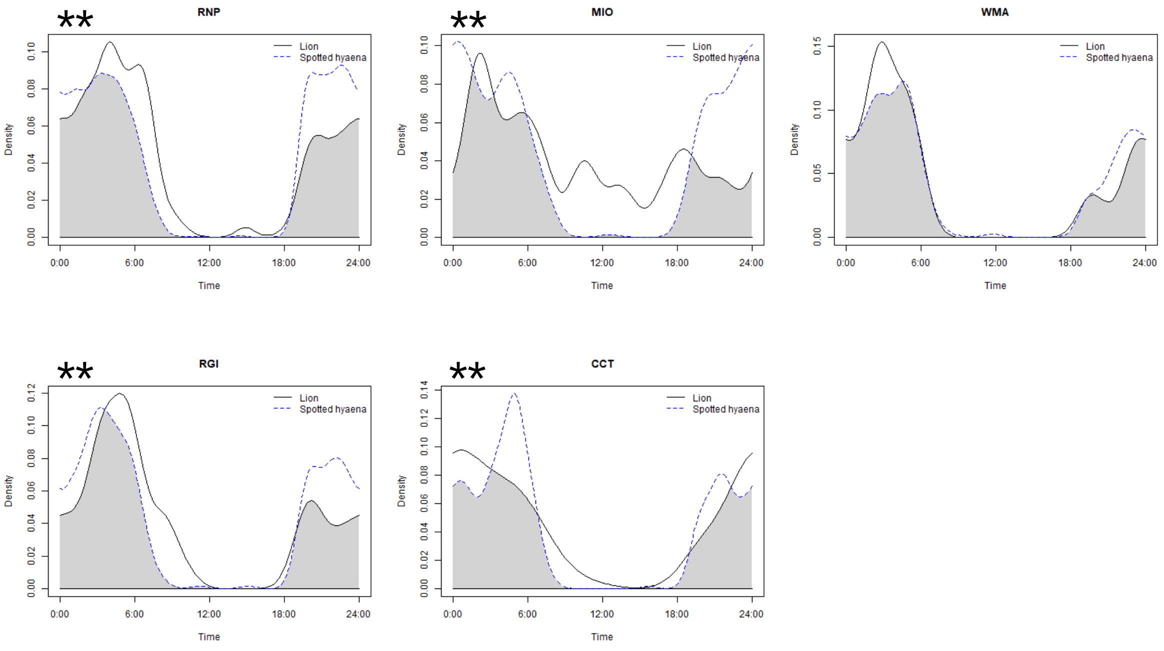

(F) Lion – Striped hyaena

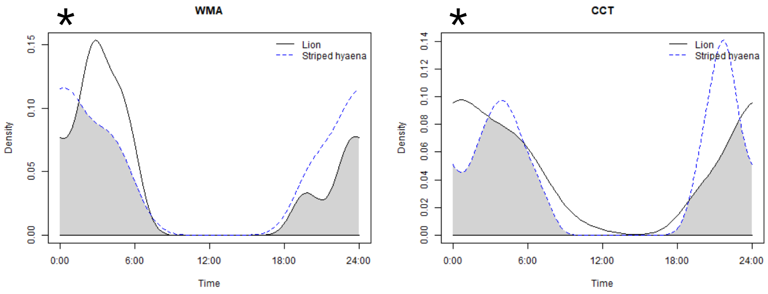

(G) Lion – African wild dog

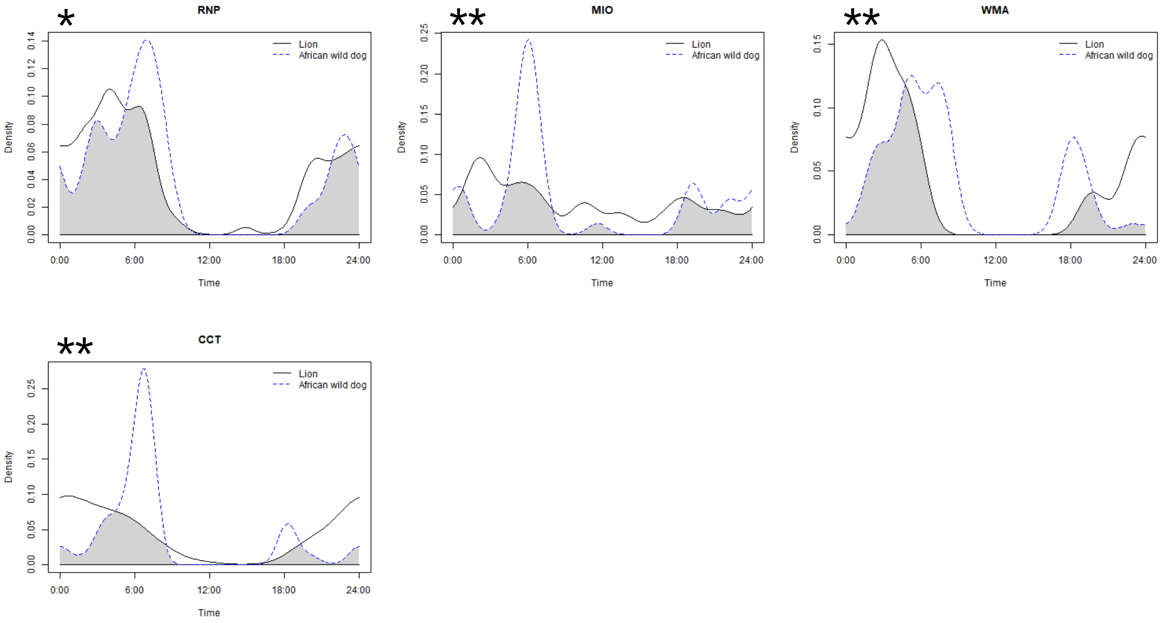

(H) Spotted hyaena – Striped hyaena

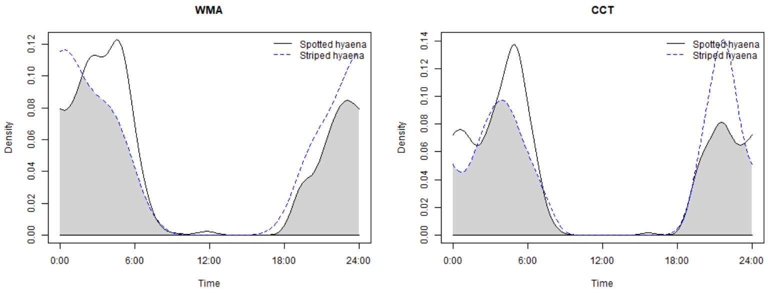

(I) Spotted hyaena – African wild dog

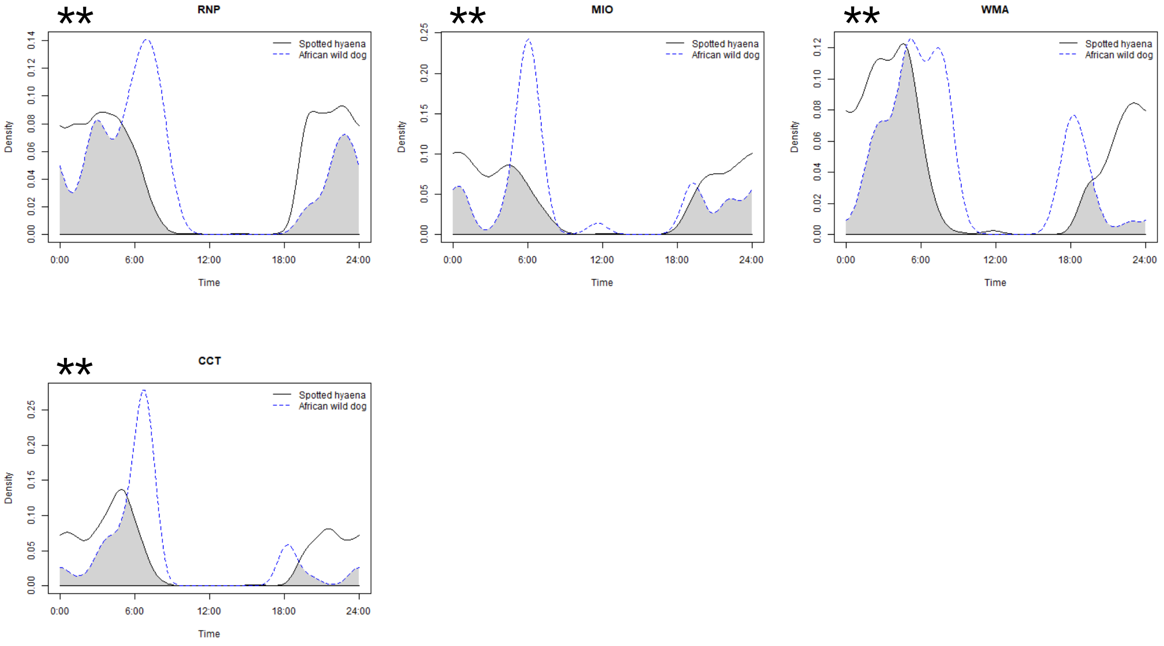

(J) Striped hyaena – African wild dog

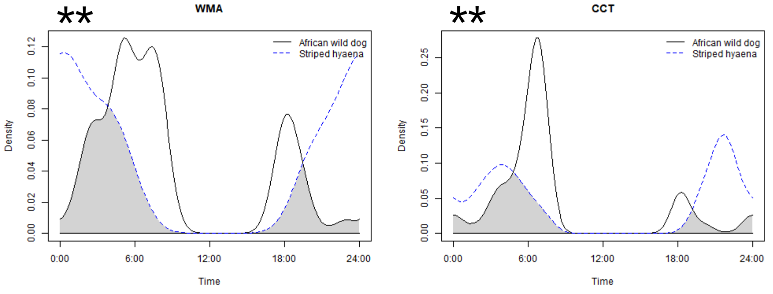

(K) Leopard (M) – Leopard (F)

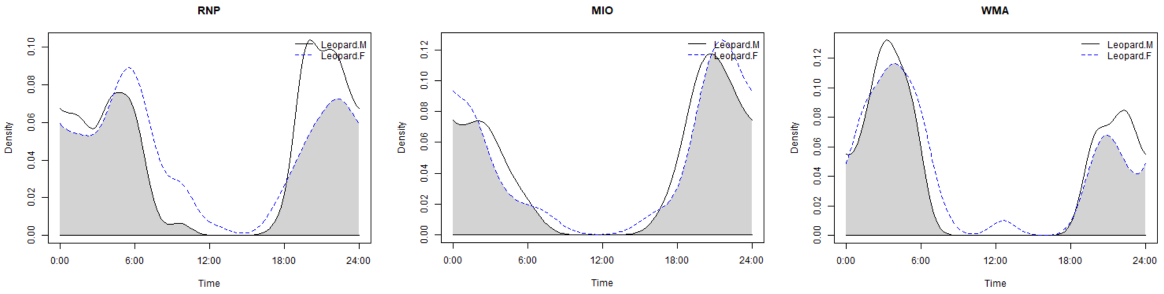

(L) Lion (M) – Lion (F)

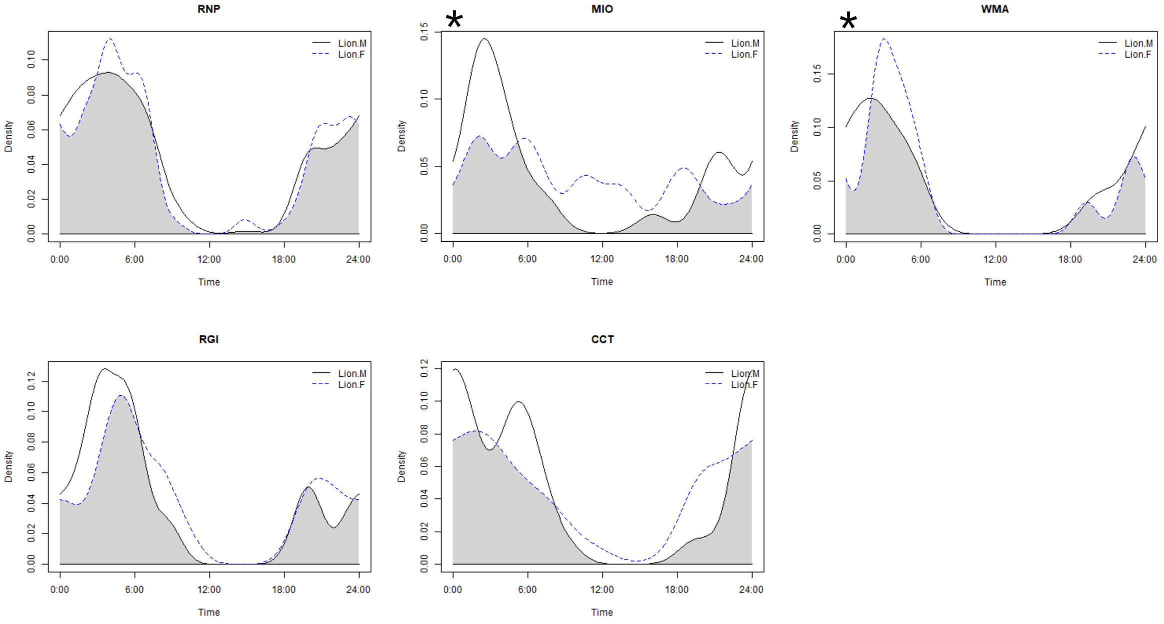

Supplement: S6 File — (PDF) [file pone.0256876.s006.pdf]
