# Supplementary material for: Temporal partitioning and spatiotemporal avoidance among large carnivores in a human-impacted African landscape
Source: PLoS One. 2021 Sep 10;16(9):e0256876. doi: 10.1371/journal.pone.0256876 (PMC8432863; doi:10.1371/journal.pone.0256876)

S7 Full temporal spacing analysis results

Figure S7.1 (overleaf): Full temporal spacing analysis results. Left-hand plots show the detection probability of species B before and after a capture of species A (at hour 0) at the same station. Boxplots show the expected probability of detecting species B in each time unit before and after capture of species A if there were no relationship between detections of species A and species B, while dots indicate the observed detection probability of species B for each time unit before and after capture of species A. Expected detection probabilities were derived by randomly sampling 1000 times from the observed activity pattern probability density function for that species. Right-hand plots show p-values for each time unit; bars below the blue line indicate time units where the observed probability of detecting species B differed significantly from the expected probability of detection ($p < .05$).

(A) 6 hours before and after capture, in units of 30 minutes

Species A: lion, Species B: leopard

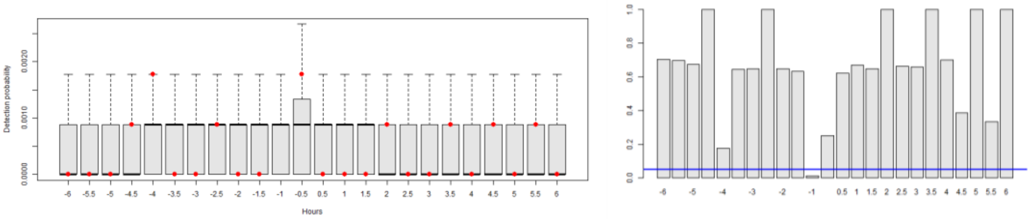

Species A: lion, Species B: spotted hyaena

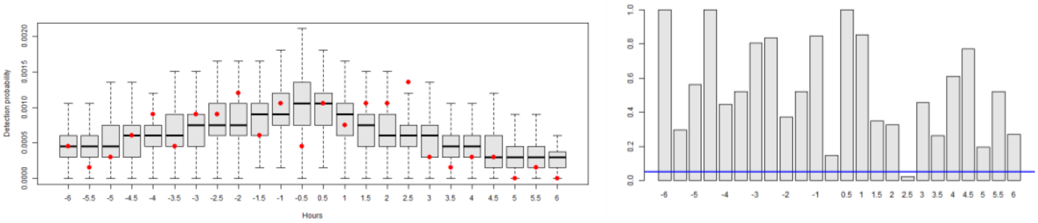

Species A: spotted hyaena, Species B: lion

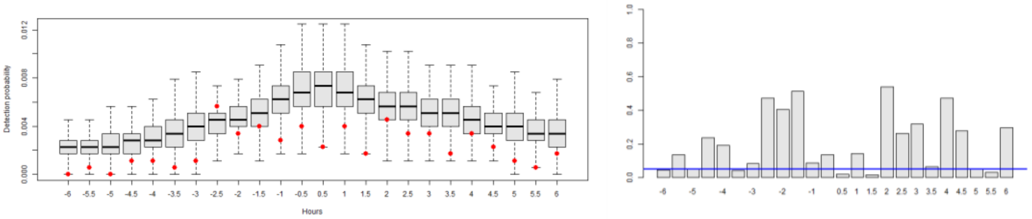

Species A: spotted hyaena, Species B: leopard

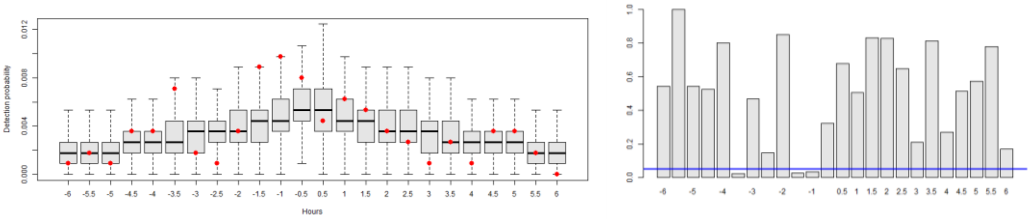

Species A: leopard, Species B: lion

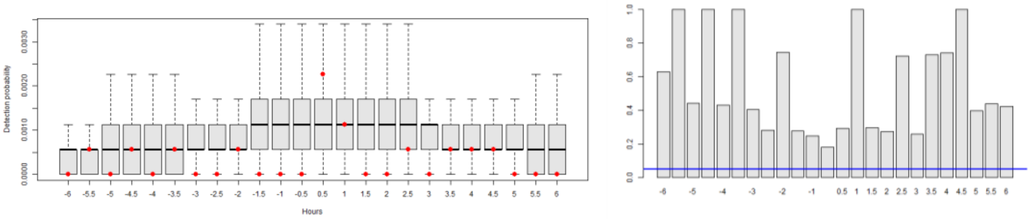

Species A: leopard, Species B: spotted hyaena

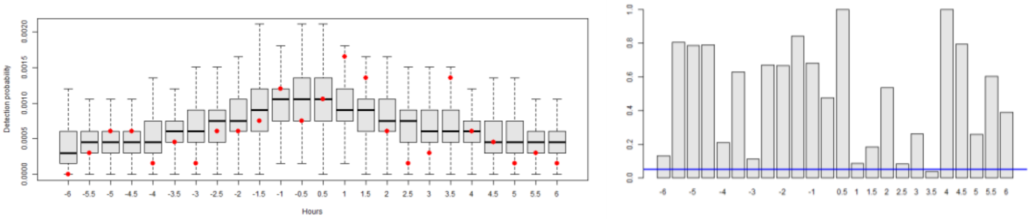

(B) 12 hours before and after capture, in units of 1 hour

Species A: lion, Species B: leopard

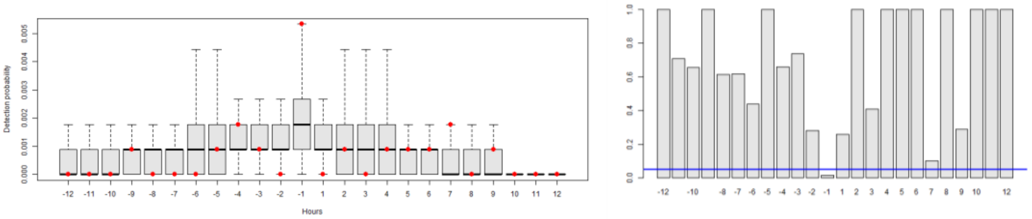

Species A: lion, Species B: spotted hyaena

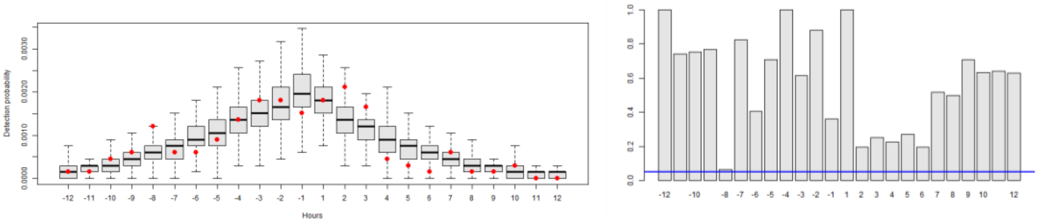

Species A: spotted hyaena, Species B: lion

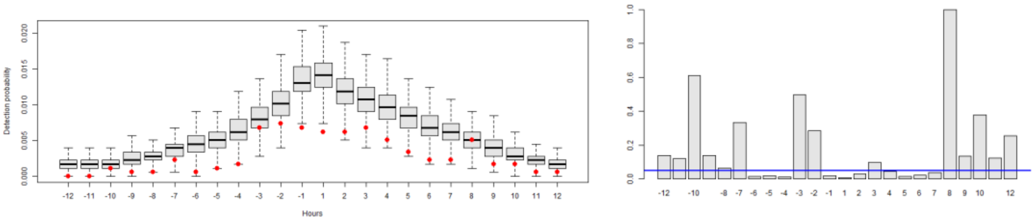

Species A: spotted hyaena, Species B: leopard

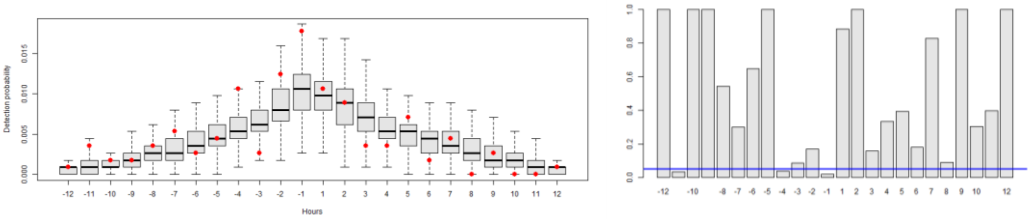

Species A: leopard, Species B: lion

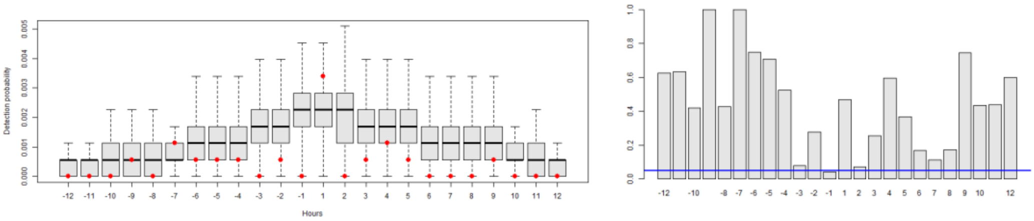

Species A: leopard, Species B: spotted hyaena

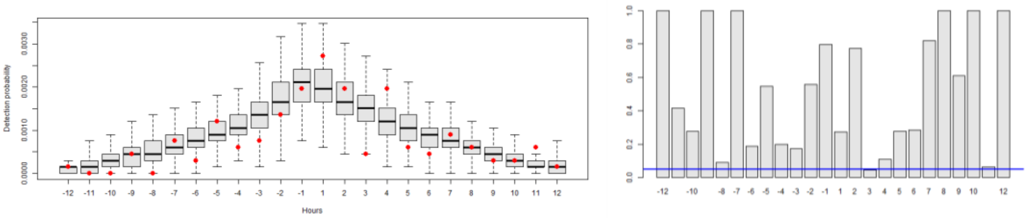

(C) 48 hours before and after capture, in units of 6 hours

Species A: lion, Species B: leopard

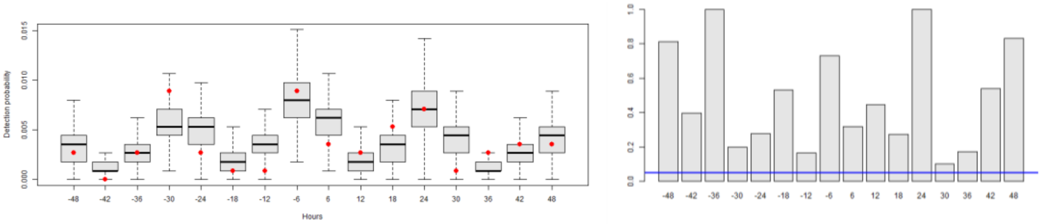

Species A: lion, Species B: spotted hyaena

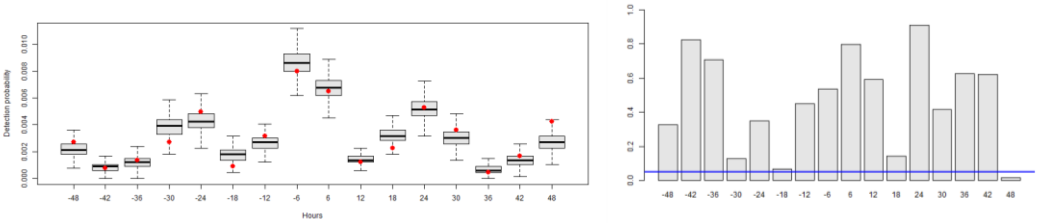

Species A: spotted hyaena, Species B: lion

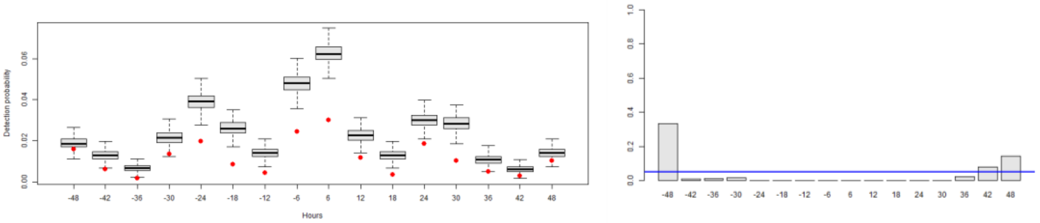

Species A: spotted hyaena, Species B: leopard

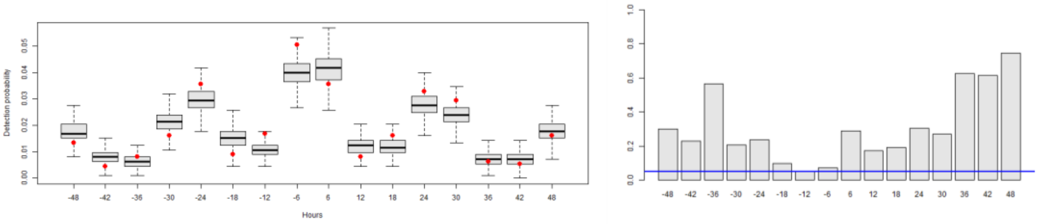

Species A: leopard, Species B: lion

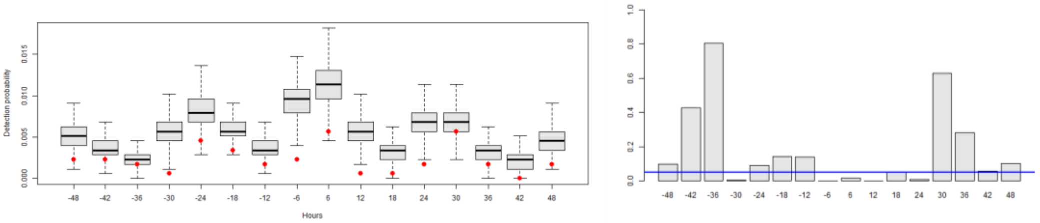

Species A: leopard, Species B: spotted hyaena

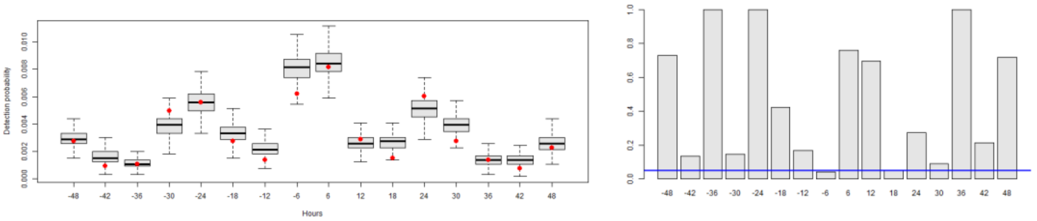

Supplement: S7 File — (PDF) [file pone.0256876.s007.pdf]
