# Supplementary material for: Temporal partitioning and spatiotemporal avoidance among large carnivores in a human-impacted African landscape
Source: PLoS One. 2021 Sep 10;16(9):e0256876. doi: 10.1371/journal.pone.0256876 (PMC8432863; doi:10.1371/journal.pone.0256876)

S8 Ruaha-Rungwa large carnivore population density estimates

Table S8.1: Population density estimates for leopard (Searle et al. 2021), lion (Strampelli et al. in prep.), spotted hyaena (Searle et al. in prep.), and striped hyaena (Hardouin et al. 2021). Estimates were produced via spatially explicit capture-recapture (SECR) modelling of data from the systematic camera trap surveys in Ruaha NP, MBOMIPA WMA, and Rungwa GR.

	Population density[1]			
Survey site	Leopard	Lion	Spotted hyaena	Striped hyaena
Ruaha NP *Acacia-Commiphora*	6.81 ± 1.24	6.12 ± 0.94	10.8 ± 1.08	N/A
Ruaha NP miombo woodland	3.23 ± 1.25	1.75 ± 0.62	3.55 ± 0.72	N/A
MBOMIPA WMA *Acacia-Commiphora*	4.23 ± 1.02	4.06 ± 1.03	5.11 ± 0.81	1.36 ± 0.50
Rungwa GR miombo woodland	3.36 ± 1.09	2.25 ± 0.52	5.89 ± 0.75	N/A

[1] Individuals per 100 km^2

Figure S8.1: Plot of leopard, lion, and spotted hyaena population density estimates across the four survey sites in Ruaha-Rungwa.

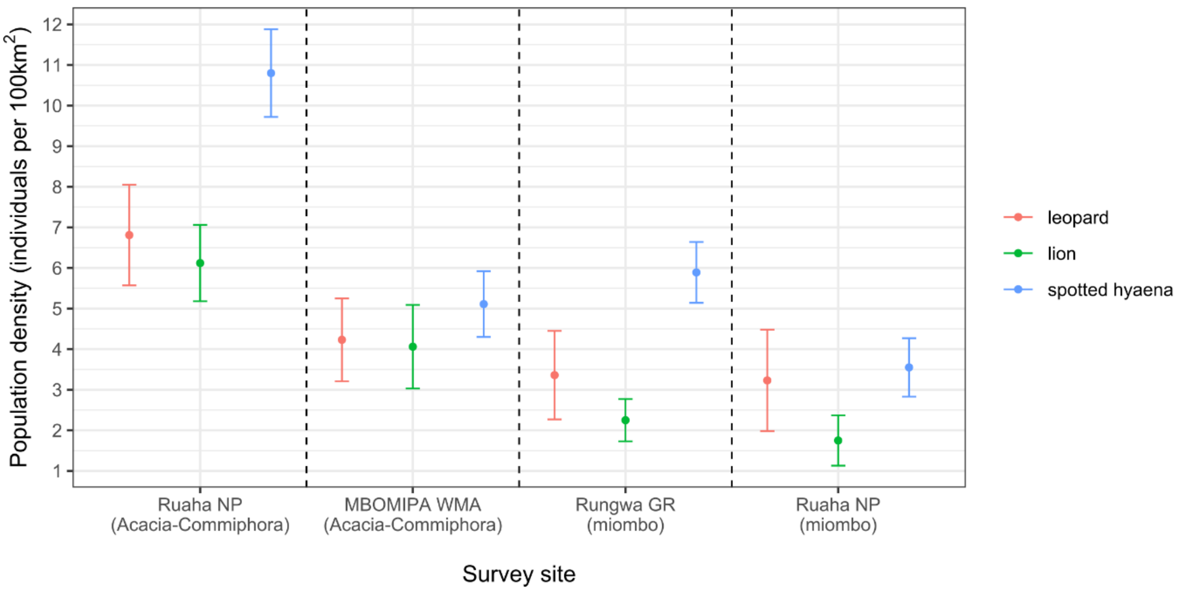

Supplement: S8 File — (PDF) [file pone.0256876.s008.pdf]
