# Supplementary material for: Temporal partitioning and spatiotemporal avoidance among large carnivores in a human-impacted African landscape
Source: PLoS One. 2021 Sep 10;16(9):e0256876. doi: 10.1371/journal.pone.0256876 (PMC8432863; doi:10.1371/journal.pone.0256876)
Supplement: Attachment — Submitted filename: Large carnivore interactions_Response to reviewers.pdf [file pone.0256876.s009.pdf]

25th July 2021

Response to reviewer comments on manuscript PONE-D-21-15323: Temporal partitioning and spatiotemporal avoidance among large carnivores in a human-impacted African landscape

We thank the Editor and the two Reviewers for their valuable comments on our submission, which we believe have measurably improved our manuscript. Below, we provide a point-by-point response to each of the issues raised by the Editor and Reviewers, detailing the changes made to our submission in light of their comments. Where relevant, we refer to the position of changes in text with the line on which it can be found in the revised version of the manuscript.

Please note that we have made some slight modifications to the authorship of the manuscript. The former second author (Jeremy J. Cusack) and third author (Josephine B. Smit) have been switched, in recognition of the slightly greater contribution of Josephine B. Smit, who is now the second author. A middle initial has also been added for Josephine B. Smit, who was previously listed as Josephine Smit.

Journal requirements

1. *Please ensure that your manuscript meets PLOS ONE's style requirements, including those for file naming.*

    We have made the following modifications to our manuscript, in order to meet *PLOS ONE*'s style requirements: we have updated the author affiliations to use numbers rather than letters; updated the formatting and levels of our headers; removed information on funding from the acknowledgements; updated our referencing to be in the style of *PLOS ONE*; modified our tables so that the title is in bold above the table, and the legend is below the table; and ensured our supporting information files are named in line with *PLOS ONE* specifications.

2. *We note that Figures 1 and S5 in your submission contain map images which may be copyrighted […]*

    The maps in Figures 1 and S5 were produced in QGIS by the lead author (Charlotte E. Searle), and are not subject to copyright. The map elements showing country borders, protected areas, and human settlements originated from shapefiles that are not subject to copyright.

    The rivers layer used in the main map in Figure 1 was modified from shapeflies obtained from Natural Earth. As declared on the Natural Earth website, "*All versions of Natural Earth raster + vector map data found on this website are in the public domain. You may use the maps in any manner, including modifying the content and design, electronic dissemination, and offset printing. The primary authors, Tom Patterson and Nathaniel Vaughn Kelso, and all other contributors renounce all financial claim to the maps and invites you to use them for personal, educational, and commercial purposes. No permission is needed to use Natural Earth. Crediting the authors is unnecessary*".

The shaded terrain basemap used in the inset map of Tanzania in Figure 1 was obtained from ESRI. As declared on the ESRI website, "*Esri grants the recipient of the Esri information contained within the esri.com Web site the right to freely reproduce, redistribute, rebroadcast, and/or retransmit this information for personal, noncommercial purposes, including teaching, classroom use, scholarship, and/or research, subject to the fair use rights enumerated in sections 107 and 108 of the Copyright Act (Title 17 of the United States Code). All copies, whether in whole or in part, shall include the appropriate Esri copyright notice*".

In line with this, we have amended the caption for Figure 1 as follows:

> Figure 1: Left: the location of the Ruaha-Rungwa landscape within Tanzania (World Terrain Base by Esri, USGS, & NOAA). Right: detail of the four systematic camera trap grids, comprising a total of 151 camera pairs, and the 56 unpaired cameras deployed in village land as part of a community camera-trapping programme (made with Natural Earth). Systematic camera trap grids were located in (1) Ruaha NP Acacia-Commiphora habitat, (2) Ruaha NP miombo habitat, (3) MBOMIPA WMA Acacia-Commiphora habitat, and (4) Rungwa GR miombo habitat.

3. *Please review your reference list to ensure that it is complete and correct.*

We have reviewed our reference list, and confirm that it is complete and correct.

Reviewer 1

This study uses camera trap data to examine the effects of human impact on temporal and spatiotemporal associations between some of Tanzania's large carnivores. The authors found evidence of avoidance and attraction between the various carnivores, and that both leopard and lion altered these behaviours in the areas with high human impact. This suggests that, at least for road-travelling behaviours, human activity may interfere with spatiotemporal partitioning mechanisms that carnivore guilds use to facilitate coexistence with one another.

This is a very well written manuscript that clearly describes its methodology and findings. The authors are also transparent about the limitations of their data and the implications of their results. I enjoyed reading this paper, and I only have one very minor suggestion which the authors can take or leave as desired. I recommend this paper be accepted for publication.

Multi-panelled figures 2 & 3: the axes titles and labels for these figures is a bit small and hard to read. I suggest increasing the font size here. To help avoid the figures becoming overly crowded, I suggest that instead of having each figure panel's axes titled individually, instead have a larger axes title placed in a central position between the panels (since in each figure, the x and y axis for the panels is the same).

We thank the Reviewer for their positive comments. We also appreciate their helpful suggestions to improve the legibility of Figures 2 and 3. In line with the recommendations, we have increased the axis font size and reduced the number of times the axis titles are repeated.

Reviewer 2

Increase in anthropogenic impacts have been resulted in disruption of ecological process across the world and competitive interactions among large carnivores are one. Present study able to depicts how human disturbance can disrupt inter-predator relationship. Following are the comments from me:

Introduction of the manuscript is robust but have put to much emphasis on the literature of competitive interactions on large carnivores and less emphasis on how human disturbance impact these competitive relationships except one paragraph.

We acknowledge that our introduction focuses more heavily on the nature of competitive interactions among large carnivores than on how human disturbance can affect these interactions, but this was necessary for multiple reasons. First, understanding the nature of interactions among African large carnivores is an important foundation for understanding the impacts of humans on these relationships. Second, there is a substantially larger body of literature available on the topic of species interactions, due to the emerging nature of research into human impacts on these interactions.

We have added a sentence to the introduction acknowledging the relative lack of research on the topic of human impacts on large carnivore interactions (lines 99-102), to make it clear why we provide less background on this topic. This also links in with our statement at the start of our final paragraph in the introduction, that there is a need to understand how coexistence mechanisms employed by these species vary across modern, human-shaped landscapes, and how they may be affected by anthropogenic impacts. While in the original version of the manuscript this sentence implied that there is a research gap, by addressing the Reviewer's comments we now feel this gap is explicitly acknowledged.

Also, introduction lack References at certain statement. For example, Line 48, 49, 50 involves description of competition lack any reference to support the statement.

We have added the following references in support of our sentence about the different forms of competition experienced by carnivores on lines 48-50 (now lines 50-52): Amorós et al., 2020; Everatt et al., 2019; Linnell & Strand, 2000.

Another statement in line 72 and 73 i.e., it has been theorised that the species may be forced to share space in more anthropogenically impacted areas. Does this statement can be applied in protected area which lack much human disturbance and leopards rely much on cover rather than human impacted area?

We believe that the statement about lion and leopard potentially being forced to share space in anthropogenically-impacted areas is highly relevant to our study sites, including those in protected areas. Our data were obtained from across an anthropogenic impact gradient – including completely unprotected village land, a protected but impacted area (Wildlife Management Area), a protected area with extractive activities (Game Reserve), and a protected area with non-extractive activities (National Park). Despite their status – and like many

protected areas across Africa – the protected areas in our study landscape are all still subject to human impacts, such as bushmeat poaching near boundaries and in more remote areas, albeit at different levels related in part to the strength of protection in place. Indeed, the study we refer to here was conducted inside a strictly-protected area – Mozambique's Limpopo National Park – which is nonetheless impacted by subsistence farming and bushmeat poaching (Strampelli et al., 2018).

Under data collection heading paragraph includes line number from 136-142 is confusing. For example, in line number 136 i.e., We used data from camera trap surveys at four sites in the Ruaha-Rungwa landscape (38), what does 38 means is not clear.

We have modified our phrasing in section *2.3. Data collection*, to improve the section's clarity. Specifically, we have added further information alongside reference 38 (now reference 40), stating that this refers to a publication which provides further detail on the survey design (lines 147-148).

In Analysis section from line number 227 to 234 not a single reference has been provided to support that this analysis has been used in some earlier study. In survey effort a total independent capture of the species under study has been provided since study studies at different sites hence independent captures at different sites should also mentioned in survey effort.

In section *2.4.2. Spatiotemporal avoidance and attraction*, we specifically state that we used the temporal spacing analysis developed by Cusack et al. (2017; now reference 46) to investigate whether large carnivores exhibited intra-guild spatiotemporal avoidance or attraction at stations where they co-occurred (lines 217-219). The procedure that follows – including the steps described on lines 227-234 (now lines 233-240) and flagged by the Reviewer – is as described in this original study. By stating that we used the analysis developed in the referenced study at the start of the section, we hope it is sufficiently clear that all subsequent steps described are as set out in the referenced study.

Also, how much captures of male and female leopards were got has not mentioned in survey effort which also need to be stated.

We do provide information on the number of independent captures at different sites, including a breakdown of the number of captures of males, females, and individuals of unknown sex for lion and leopard, in Table 1, which is referred to in section *3.1. Survey effort*. We feel that also including this information in-text in this section would be needlessly repetitive, but would be willing to make this change if the Editor considers it necessary.

Authors have able to justified their findings thoroughly in discussion. But discussion lack light on spatio-temporal attraction in relation to different habitat which authors have sampled in RNP. Do these habitats differ in their structure and visibility which could further affect the outcome of the competitive interactions?

Although the overall habitat type differed between our study sites (miombo woodland, *Acacia-Commiphora* bushland), vegetation structure and visibility also varied among individual stations within each survey site. Each grid had camera stations located in more open areas where competitors are likely to be more visible to one another – such as grassland areas within *Acacia-Commiphora* habitat and *mbugas* (drainage lines) within miombo woodland – and stations located in more dense areas, where fine-scale competitor avoidance may be more easily achieved. As such, while we agree with the Reviewer that the role of vegetation structure on competitive interactions among large carnivores would be an interesting avenue for research, we do not feel it is possible for us to make any inferences about this on the basis of our analysis.

References

Amorós, M., Gil-Sánchez, J. M., López-Pastor, B. de las N., & Moleón, M. (2020). Hyaenas and lions: How the largest African carnivores interact at carcasses. *Oikos*, *129*(12), 1820–1832. https://doi.org/10.1111/oik.06846

Cusack, J. J., Dickman, A. J., Kalyahe, M., Rowcliffe, J. M., Carbone, C., MacDonald, D. W., & Coulson, T. (2017). Revealing kleptoparasitic and predatory tendencies in an African mammal community using camera traps: a comparison of spatiotemporal approaches. *Oikos*, *126*(6), 812–822. https://doi.org/10.1111/oik.03403

Everatt, K. T., Moore, J. F., & Kerley, G. I. H. (2019). Africa's apex predator, the lion, is limited by interference and exploitative competition with humans. *Global Ecology and Conservation*, *20*, e00758. https://doi.org/10.1016/J.GECCO.2019.E00758

Linnell, J. D. C., & Strand, O. (2000). Interference interactions, co- existence and conservation of mammalian carnivores. *Diversity and Distributions*, *6*(4), 169–176. https://doi.org/10.1046/J.1472-4642.2000.00069.X

Strampelli, P., Andresen, L., Everatt, K. T., Somers, M. J., & Rowcliffe, J. M. (2018). Habitat use responses of the African leopard in a human-disturbed region of rural Mozambique. *Mammalian Biology*, *89*, 14–20. https://doi.org/10.1016/J.MAMBIO.2017.12.003
